# Prevalence, persistence, clearance and risk factors for HPV infection in rural Uyghur women in China

**DOI:** 10.1186/s12905-023-02558-y

**Published:** 2023-08-15

**Authors:** Gulixian Tuerxun, Guligeina Abudurexiti, Guzalinuer Abulizi

**Affiliations:** 1 Fifth Department of Gynecologic Surgery, Xinjiang Medical University Affiliated Tumor Hospital, Urumqi, 830000 China; 2 Xinjiang Key Laboratory of Oncology, No. 789 East Suzhou Street, Xinshi District, Urumqi, 830000 China

**Keywords:** HPV, Prevalence, Persistent infection, Risk factor, Uyghur women

## Abstract

**Background:**

The incidence of cervical cancer in Uyghur women ranks first among those in Han and other ethnic minority groups. We aimed to understand the natural history of HPV in Uyghur women.

**Methods:**

A longitudinal cohort study on the natural history of HPV infection in rural Uyghur women in China was conducted between May 2013 and May 2014. A total of 11000 women from South Xinjiang underwent HPV screening by careHPV and liquid-based cytology. Ultimately, a total of 298 women with positive HPV and normal biopsy results or CIN1 were enrolled to participate in a study including follow-up HPV testing for two years.

**Results:**

The HPV infection rate in Uyghur women was 9.15%. Among the participants, the careHPV test showed that 298 women were HPV-positive, and histology showed CIN1 or normal results for these women at baseline. Among these patients, after 24 months of initial recruitment, 92 (30.87%) patients had persistent HPV infections, and 206 (69.13%) had cleared HPV infection. Univariate analysis showed that persistent HPV infection was associated with age and shower frequency (*P* < 0.001 and *P* = 0.047, respectively).

**Conclusions:**

Our results suggest that women over the age of 50 years who have been infected with HR-HPV for more than 1 year should be regularly screened and monitored for HPV. In addition, education should be strengthened to improve poor health habits in these women.

## Background

High-risk human papillomavirus (HR-HPV) is an important cause of cervical cancer. Fortunately, most genital HPV infections are transient, with approximately 90% of women spontaneously clearing infections within 12–24 months of infection [1]. Persistent HR-HPV infection increases the risk of cervical precancerous lesions [2].

According to China's cancer statistics in 2015, the incidence of cervical cancer has increased significantly in China compared with developed countries. This may reflect the inadequacy of Papanicolaou (Pap) screening and the increasing prevalence of HPV infection in mainland China [3].

The incidence of cervical cancer in Uyghur women ranges from 459/100,000 to 590/100,000, which is the highest in China. Moreover, the age of HPV infection in Uyghur women is earlier than that among women in other parts of China [4]. However, some surveys of HPV infection showed that HPV infection rates were lower in Uyghur women than those among women of other nationalities in China [5]. Data on the natural history of HPV infection in Uyghur women are limited. As reported in this paper, we conducted a population-based study that included 11,000 Uyghur women, assessed the prevalence of HPV infection in Uyghur women, and identified risk factors associated with HPV infection.

## Material and methods

### Patients

In total, 6,000 volunteers from Hotan and 5,000 volunteers from Kashgar, Xinjiang, China, where the incidence of cervical cancer is high, were enrolled in a population-based study between May 2013 and May 2014.

The number of subjects is directly proportional to the population of the county. The inclusion criteria were as follows: Participants aged 20 years or older; those who had not received the HPV vaccine; those who had a complete uterus; those were not pregnant; and those who had no history of invasive cervical cancer. The research ethics committee of the Affiliated Tumor Hospital of Xinjiang Medical University approved the research program. All methods were performed in accordance with the relevant guidelines and regulations. Each participant signed an informed consent form. For participants who were illiterate, informed consent was obtained from a parent or legal guardian.

All participants completed a structured questionnaire based on potential risk factors, including age category, education level, occupation type, sexual behavior, birth history, age at first sexual intercourse, the use of contraception methods and personal hygiene. Each participant underwent a pelvic exam and two cervical swabs. Cervical specimens were collected using a broom device placed in liquid-based cytology medium (Triplex International Bioscience [China] Co. Ltd.). The sample collection brush was used for HPV testing (care HPV ^TM^, Qiagen Inc.). Among these patients, those with positive results (TCT ≥ ASCUS or HPV-positive) were referred for colposcopy. Following histology, patients with grade 2 cervical intraepithelial neoplasia (CIN 2) or CIN2 + were referred for further treatment.

### Follow-up

Women with normal cervical histology and CIN1 were included in the follow-up survey. A follow-up of 2 years was scheduled every 12 months. During the follow-up period, each participant underwent a pelvic exam and specimen collection for HPV testing as described above.

### The careHPV test

Fourteen HR-HPVs (including 16, 18, 31, 33, 35, 39, 45, 51, 52, 56, 58, 59, 66 and 68) were detected in cervical specimens through careHPV test ,which is an in vitro nucleic acid hybridization assay coupled with signal amplification that uses microplate chemiluminescence for the qualitative detection of HPV [6]. Samples were detected according to the manufacturer’s instructions. Cytology results were classified according to the 2001 Bethesda system.

### Colposcopy

After collecting cervical cell specimens, colposcopy was performed to determine whether the women had positive HPV or Thinprep Cytology Test (TCT) results, and a direct biopsy or four-quadrant biopsy was performed. Cervical curettage was performed when necessary. Colposcopy was performed according to general guidelines.

### Definitions of HPV infection persistence and clearance

Persistent HR-HPV infection was defined as the detection of HR-HPV positivity at enrollment and follow-up (12-month and 24-month follow-ups). Clearance was defined as HR-HPV positivity at enrollment but negativity at follow-up.

### Statistical analysis

The data were analyzed by SPSS 25.0, and the count data are expressed by the frequency and rate. The relationship between the rate of HPV infection (persistent HPV infection) and related factors was analyzed by binary logistic regression analysis. The related factors were screened by single-factor binary logistic regression analysis, and then logistic regression models were established by the entry method and stepwise regression method to explore the related factors. The test level was α = 0.05.

## Results

### Characteristics of the study cohort

From May 2013 to May 2014, a total of 11,000 women aged 20–69 years in Hotan and Kashgar were recruited for cohort studies. The sociodemographic characteristics of the participants are shown in Table 1. The average age of the participants was 38.93 ± 9.74 years, and women aged 40–49 years accounted for 33.72% of the total sample. More than 80% of the participants were farmers, and almost 70% were illiterate or had only a primary school education. The participants preferred intrauterine devices (60.6%) for contraception, and more than 60% got married and gave birth before 18 years of age (Table 1).

**Table 1:** Demographic characteristics of Uyghur women screened for cervical cancer (*n* = 11000)

Characteristic	N	%
**Age (years)**		
20–29	2115	19.23
30–39	3562	32.38
40–49	3709	33.72
≥ 50	1614	14.67
**Educational level**		
Illiterate and Primary	7671	69.74
Secondary and Higher	3329	30.26
**Occupation type**		
Farmer	9733	88.48
Nonfarmer	1267	11.52
**Age at first marriage (years)**		
< 18	7194	65.4
≥ 18	3806	34.6
**Age at first birth (years)**		
< 18	3018	27.44
≥ 18	7551	72.56
**Number of marriages**		
≤ 2	8733	79.39
> 2	2267	20.61
**Number of pregnancies**		
≤ 3	4798	43.62
> 3	6202	56.38
**Number of childbirths**		
≤ 3	7487	68.06
> 3	3513	31.94
**Contraception method**		
IUD	6662	60.56
Coitus interruptus	74	0.67
OC	747	6.79
Condom	870	7.9
Sterilization	988	8.89

### Risk factors for HPV infection

Of the 11,000 women surveyed, 1,006 women had HPV infection. The HPV infection rate was 9.15%. Table 2 summarizes the prevalence of HPV infection based on sociodemographic and behavioral characteristics. In particular, the results showed that HPV infection rates were significantly higher in participants aged 50 years or older than in those under 50 years of age (*P* = 0.000, OR = 1.802), whereas educational level was not significantly.associated with HPV infection. Nonfarmers had a higher HPV infection rate than farmers (*P* = 0.004, OR = 1.320). Women who gave birth for the first time over the age of 18 years had a lower HPV infection rate than those who gave birth for the first time under the age of 18 years (*P* = 0.002, OR = 0.802). Risk was not associated with the number of pregnancies, number of childbirths, age at first marriage or number of marriages. Women with a higher sex frequency had a higher rate of HPV infection (*P* = 0.000, OR = 1.694; *P* = 0.000, OR = 1.539). Compared to women who did not use IUDs and OCs, those who did use them showed a lower rate of HPV infection (*P* = 0.000, OR = 0.726; *P* = 0.017, OR = 0.698). Regarding shower frequency, the higher the shower frequency was, the lower the HPV infection rate (*P* = 0.000, OR = 0.608).

**Table 2:** Sociodemographic Characteristics and Behaviors of Women with HPV Infection (*n* = 11000)

	N	HPV-positive (n)	HPV-positive (%)	OR (95% CI)	P
**Age (years)**					
20–29	2115	167	7.9	1.000	
30–39	3562	311	8.7	1.116(0.917–1.358)	0.273
40–49	3709	312	8.4	1.071(0.881–1.303)	0.491
≥ 50	1614	216	13.4	1.802(1.456–2.231)	0.000
**Educational level**					
Illiterate and Primary	7671	692	9.0	1.000	
Secondary and Higher	3329	314	9.4	1.05(0.913–1.208)	0.492
**Occupation type**					
Farmer	9733	862	8.9	1.000	
Nonfarmer	1267	113	11.4	1.320(1.094–1.591)	0.004
**Age at first marriage**					
< 18	7194	671	9.3	1.000	
≥ 18	3806	335	8.8	0.938(0.818–1.076)	0.363
**Age at first birth**					
< 18	3018	318	10.5	1.000	
≥ 18	7551	652	8.6	0.802(0.697–0.924)	0.002
**Number of marriages**					
≤ 2	8733	775	8.9	1.000	
> 2	2267	231	10.2	1.165(0.998–1.36)	0.053
**Number of pregnancies**					
≤ 3	4798	422	8.8	1.000	
> 3	6202	584	9.4	1.078(0.945–1.229)	0.263
**Number of childbirths**					
≤ 3	7487	672	9.0	1.000	
> 3	3513	334	9.5	1.065(0.928–1.223)	0.367
**Intercourse frequency**					
≤ once a week	3152	204	6.5	1.000	
2–3 times a week	5385	565	10.5	1.694(1.434–2.001)	0.000
≥ 4 times a week	2463	237	9.6	1.539(1.266–1.870)	0.000
**Contraception method**					
IUD	6662	538	8.1	0.726(0.638–0.828)	0.000
Coitus interruptus	74	6	8.1	0.876(0.379–2.023)	0.756
OC	747	50	6.7	0.698(0.52–0.937)	0.017
Condom	870	74	8.5	0.917(0.716–1.175)	0.495
Sterilization	988	97	9.8	1.090(0.875–1.359)	0.442
**Shower frequency (days between showers)**					
≥ 7 days	1937	220	11.4	1.000	
3–5 days	4748	474	10.0	0.866(0.731–1.025)	0.095
1–3 days	4315	312	7.2	0.608(0.507–0.729)	0.000

The multivariate logistic regression analysis showed that the following characteristics were significantly associated with HPV infection (*P* < 0.05) (Table 3): (a) being aged 50 years or older (OR = 1.688); (b) being a nonfarmer (OR = 1.254); (c)giving birth for the first time under 18 years of age (OR = 0.789); (d) having frequent intercourse (OR = 1.607; OR = 1.426); (e) showering less frequently (OR = 0.789; OR = 0.593); and (f) using IUDs or OCs (OR = 0.683; OR = 0.668).

**Table 3:** Multivariate Logistic Regression Analysis of the Factors Related to HPV Infection (*n* = 11000)

Variable	Model 1		Model 2	
	*OR*(95% *CI*)	*P*	*OR*(95% *CI*)	*P*
**Age (years)**				
20–29	1.000		1.000	
30–39	1.07(0.874–1.309)	0.514	1.07(0.874–1.309)	0.514
40–49	1.017(0.83–1.247)	0.869	1.017(0.83–1.247)	0.869
≥ 50	1.688(1.348–2.113)	0.000	1.688(1.348–2.113)	0.000
**Occupation type**				
Farmer	1.000		1.000	
Nonfarmer	1.254(1.03–1.527)	0.024	1.254(1.03–1.527)	0.024
**Age at first birth (years)**				
< 18	1.000		1.000	
≥ 18	0.789(0.683–0.911)	0.001	0.789(0.683–0.911)	0.001
**Intercourse frequency**				
≤ once a week	1.000		1.000	
2–3 times a week	1.607(1.344–1.922)	0.000	1.607(1.344–1.922)	0.000
≥ 4 times a week	1.426(1.161–1.752)	0.001	1.426(1.161–1.752)	0.001
**Contraception method**				
IUD	0.683(0.596–0.783)	0.000	0.683(0.596–0.783)	0.000
OC	0.668(0.495–0.902)	0.008	0.668(0.495–0.902)	0.008
**Shower frequency (days between showers)**				
≥ once a week	1.000		1.000	
Once every 3–5 days	0.789(0.659–0.944)	0.010	0.789(0.659–0.944)	0.010
Once every 1–3 days	0.593(0.488–0.721)	0.000	0.593(0.488–0.721)	0.000

Model 1 used the entry method; Model 2 used the stepwise method

### Assessment after 12 months and 24 months of follow-up

Among the 11,000 participants, 1006 women tested positive for HPV infection. Of these women, 429 (3.9%) showed positive cytology tests, including AGC (*n* = 33), ASC-US (*n* = 144), ASC-H (*n* = 64), LSIL (*n* = 117), HSIL (*n* = 67), and SCC (squamous cell carcinoma of the cervix) (*n* = 4). Participants who had a positive result for both tests were referred for colposcopy. Ultimately, 1380 women underwent a cervical biopsy. The results were divided into normal results, CIN1, CIN2, CIN3, SCC and adenocarcinoma, with numbers of 1064, 147, 61, 79, 26 and 2, respectively. Patients with CIN2 and CIN2 + received definitive treatment. In women with HR-HPV infection, those with CIN1 or normal results (*n* = 805) were included in the follow-up study. Of these 805 women, 320 gave consent to participate in our study. Ultimately, 298 women (93.13%) completed the survey and were tested for HPV after 12 months and 24 months of follow-up. A total of 22 women (6.87%) were lost to follow-up (2 were pregnant, 2 died, and 18 lost contact). Among the 298 HPV-positive patients with CIN1 or normal cervical histology at baseline, 120 (40.26%) had persistent infection, and 178 (59.74%) had clearance of the infection after 12 months. After 24 months, 92 (30.87%) patients had persistent HPV infection, and 206 (69.13%) had cleared HPV infection. The relationships of HPV clearance and persistence after 24 months with sociodemographic and behavioral characteristics in HPV-positive participants are described in Table 4. Our study showed that women aged 50 years or older had higher rates of persistent HPV infection than those aged under 50 years (*P* = 0.003, OR = 3.612). Women who gave birth to more than 3 children had higher rates of persistent HPV infection than those who gave birth to 3 or fewer children (*P* = 0.02, OR = 1.828). Women with less frequent showers had a higher rate of HPV infection (*P* = 0.031, OR = 0.538; *P* = 0.012, OR = 0.416). A multivariate logistic regression between sustained HPV infection and subjects’ demographic and behavioral characteristics is shown in Table 5. Characteristics significantly associated with persistent HPV infection were as follows (*P* < 0.05): (a) being aged 50 years or older (OR = 2.884) and (b) having had less frequent showers (OR = 0.579).

**Table 4:** Sociodemographic Characteristics and Behaviors of Participants with Persistent HPV Infection (*n* = 298)

	N	HPV persistent infection (n)	HPV persistent infection (%)	*OR*(95% CI)	P
**Age**					
20–29	44	10	22.7	1.000	
30–39	82	19	23.2	1.025(0.429–2.452)	0.955
40–49	106	29	27.4	1.281(0.562–2.920)	0.557
≥ 50	66	34	51.5	3.612(1.537–8.490)	0.003
**Educational level**					
Illiterate and Primary	223	70	31.4	1.000	
Secondary and Higher	75	22	29.3	0.907(0.512–1.607)	0.739
**Occupation type**					
farmer	270	83	30.7	1.000	
Nonfarmer	28	9	32.1	1.067(0.463–2.458)	0.879
**Age at first marriage (years)**					
< 18	216	71	32.9	1.000	
≥ 18	82	21	25.6	0.703(0.397–1.245)	0.227
**Age at first birth (years)**					
< 18	103	34	33.0	1.000	
≥ 18	195	58	29.7	0.859(0.515–1.435)	0.562
**Number of marriages**					
≤ 2	230	73	31.7	1.000	
> 2	68	19	27.9	0.834(0.459–1.517)	0.552
**Number of pregnancies**					
≤ 3	122	31	25.4	1.000	
> 3	176	61	34.7	1.557(0.933–2.599)	0.090
**Number of childbirths**					
≤ 3	197	52	26.4	1.000	
> 3	101	40	39.6	1.828(1.099–3.043)	0.020
**Intercourse frequency**					
≤ once a week	61	22	36.1	1.000	
2–3 times a week	176	56	31.8	0.827(0.449–1.525)	0.543
≥ 4 times a week	61	14	23.0	0.528(0.239–1.167)	0.115
**Contraception method**					
IUD	156	46	29.5	0.873(0.534–1.427)	0.588
Coitus interruptus	2	0	0.0	0.000(0.000-.)	0.999
OC	10	2	20.0	0.550(0.114–2.642)	0.455
Condom	28	9	32.1	1.067(0.463–2.458)	0.879
Sterilization	26	10	38.5	1.448(0.631–3.326)	0.383
**Shower frequency (days between showers)**					
≥ 7 days	91	38	41.8	1.000	
3–5 days	133	37	27.8	0.538(0.306–0.944)	0.031
1–3 days	74	17	23.0	0.416(0.210–0.824)	0.012

**Table 5:** Multivariate logistic regression analysis of the factors related to persistent HPV infection (*n* = 298)

Variable	Model 1		Model 2	
	*OR*(95% *CI*)	*P*	*OR*(95% *CI*)	*P*
**Age (years)**				
< 50				
≥ 50	2.741(1.528–4.916)	0.001	2.884(1.619–5.140)	< 0.001
**Shower frequency (days between showers)**				
≥ 7 days				
< 7 days	0.624(0.359–1.084)	0.094	0.579(0.338–0.994)	0.047
**Number of childbirths**				
≤ 3				
> 3	1.420(0.825–2.445)	0.206	-	-

Model 1 used the entry method; Model 2 used the step-by-step method

## Discussion

Due to its high morbidity and mortality, cervical cancer has become the most important health problem in Xinjiang, China. However, the phenomenon of lower rates of HPV infections and higher rates of cervical cancer is difficult to explain. Although most HPV infections are transient, persistent HPV infection is a strong predictor of cervical cancer, especially in women with persistent HR-HPV types. Therefore, we conclude that Uyghur women may have a longer duration of HR-HPV infection than women from other parts of China. In addition, this persistence of HPV may cause differences in the incidence of cervical cancer in Uyghur women compared with women in other parts of China.

In this study, we found that the HR-HPV infection rate was 9.15% among Uygur women, lower than that among women in other parts of China, including rates of 19.81% in Guangdong Province [7], 22.8% in Hunan Province [8], and 12.9% in Yunnan Province [9]. The prevalence of HPV among Uyghur women estimated in our study differs from a previous report, which focused on a small percentage of participants in the Cervical Screening Program [10]. The difference in HPV prevalence might also be due in part to differences in the sample population and methods used. However, our study included a large sample size, and the impact of individual factors on the overall data was determined to some extent.

Viral, host and environmental factors can affect the process of HPV infection. Since HPV infection is a sexually transmitted disease, sexually active women are generally susceptible to HPV, especially high-risk HPV. Herrero R reported that the age-specific prevalence of HPV exhibited a “U” shaped curve. The HPV infection rate was the highest at ages < 16 years, then it declined gradually, reaching the lowest at the age of 41–45 years. At ages > 50 years, it began to increase again [11]. In our study, HPV infection rates were significantly higher among women over 50 years of age than among women under 50 years of age, consistent with other reports [12, 13]. This may be because elderly women have poor immune function, which is more likely to cause persistent HPV infection. In addition, changes in hormone levels in elderly individuals lead to a decrease in HPV clearance [14]. The infection rate of nonfarmers was relatively higher than that of farmers. This might be because nonfarmers may have a more open attitude toward sexual behavior.

In addition, our results showed that early childbirth was associated with a higher rate of HPV infection. This might be related to the relatively low immune function of the reproductive tract. The cervical immune system of young women is not yet fully mature and might cause the cervix to be more vulnerable [15]. However, whether intrauterine devices can reduce the risk of cervical cancer remains controversial [16, 17]. As a highly effective, safe, preferential form of contraception, most Uygur women prefer IUDs over other methods. Castellsague X reported that IUD use might act as a protective cofactor in cervical carcinogenesis [18]. However, no association was found between IUD use and the detection of cervical HPV DNA among women without cervical cancer. A retrospective cohort study of new IUD users who had HPV infections showed that copper IUD users were more likely to clear HPV infections than LNG-IUD users. The authors suggested that the anti-inflammatory properties of LNG may inhibit HPV clearance [17]. Here, in our study, we found that compared to women who did not use IUDs and OCs, those who did use them showed a low rate of HPV infection. Since a multiple choice question regarding modes of contraception was included in our questionnaire, other forms of contraception may have been used by women who were using IUDs or OCs. Therefore, it is not possible to exclude their distorting effect. We are not able to draw a definite conclusion regarding the effects of IUDs and OCs on HPV infection because of too many confounding factors. Regarding shower frequency, participants with a higher shower frequency had a lower HPV infection rate, which indicated that women who were not sensitive to personal hygiene were at higher risk for HPV infection.

There is also no universally accepted definition of HPV persistence [19]. In 2012, the cervical cancer screening guidelines mentioned that persistent HPV infection for 1 year and 2 years strongly indicated the progression of CINIII or cervical cancer [20]. To the best of our knowledge, this study is the first systematic study of the natural history of HPV infection in Uyghur women. We analyzed 298 HPV-positive women with normal and CIN1 histology who underwent a two-year follow-up. Our study showed that the clearance rate for HPV infection at 12 months of follow-up was 59.74% and reached 69.13% at 24 months of follow-up. It is believed that HPV infection is "cleared" by more than 90% of people within 2 years. The prevalence of persistent HPV infection was 29.4% in Guangdong Province and 22.63% in Xi'an at 12 months of follow-up, which was lower than that in Uyghur [21, 22]. HPV16 is more prone to persistent infection than all other virus types [23]. Guzalnur et al. reported that HPVl6 infection was the most common in cervical cancer patients and normal controls in Uyghur, and the positive rate of HPVl6 in Uyghur was significantly higher than that in Sichuan Province [24]. We assumed that this might be one possible explanation for the higher HPV persistence rate in Uyghur than in other parts of China.

It has been reported that viral variables such as HPV genotype, multiple HPV infections, and host and environmental factors such as age, smoking, and the number of sexual partners are associated with HPV persistence [25–27]. However, the data are still inconsistent. Schmeink et al. suggested that HPV clearance was primarily associated with host immune responses or other intrinsic host factors rather than behavioral factors [28]. In our study, we found that age and shower frequency were factors associated with HPV persistence. Whether age is related to the natural history of HPV infection remains controversial. Zhang et al. found that age was associated with HPV clearance [22]. The increase in age led to a decrease in HPV clearance, which was basically similar to our results. This may be related to persistent infections leading to weakened immune responses, hormonal changes, and specific lifestyles in older women.

Our study also showed that among Uyghur women, the number of pregnancies was not associated with persistent HPV infection. In addition, women who gave birth to more than 3 children had higher rates of persistent HPV infection than those who gave birth to 3 or fewer children. However, the multivariate analysis showed that the number of childbirths was not associated with persistent HPV infection, which is consistent with reports that indicated that parity was not associated with HR HPV persistence [29, 30]. The risk of persistent HPV infection was not associated with age at first childbirth, age at first marriage or the number of marriages. However, in our study, we found that shower frequency was associated with persistent HPV infection. To the best of our knowledge, this has not been reported before. Poor sanitation can result in persistent HPV infection.

## Conclusion

In conclusion, HR-HPV persistence plays a key role in the progression of precancerous lesions and the development of cervical cancer. Therefore, the epidemiological and biological understanding of the natural history of HPV infection is critical for guiding the implementation of cervical cancer prevention and control strategies. Considering that the HPV vaccine is not yet fully accessible in rural areas of China, early intervention for high-risk factors for HPV infection and persistent HPV infection may play a certain role in cervical cancer prevention.

Furthermore, we should increase the awareness of HPV among women at high risk of infection. For women over the age of 50 years, those who have been infected with HR-HPV for more than 1 year should be screened and closely monitored. In addition, education should be strengthened to improve poor health habits among women.

## Data Availability

The datasets used and analyzed during the current study are available from the corresponding author upon reasonable request, since, except for the data analyzed in this article, other data in the questionnaire are still being summarized and have not yet been published.

## References

[1] Bosch X, Robles F, Diaz C, Arbyn M, Baussano M (2016). HPV-FASTER: broadening the scope for prevention of HPV related cancer. Nat Rev Clin Oncol.

[2] Bharti AC, Singh T, Bhat A, Pande D, Jadli M (2018). Therapeutic startegies for human papillomavirus infection and associated cancers. FrontBiosci.

[3] Chen WQ, Zheng RS, Baade PD, Zhang SW, Zeng HM (2016). Cancer statistics in China, 2015. CA Cancer J Clin.

[4] Peng YH, Lalei S, Zhou K, Wang ZH, Fang XZ (2003). Clinical analysis of cervical cancer cases. Chinese J Obstet Gynecol.

[5] Wang J, Tang DD, Wang K, Wang JL, Zhang ZX (2019). HPV genotype prevalence and distribution during 2009–2018 in Xinjiang, China: baseline surveys prior to mass HPV vaccination. BMC Womens Health.

[6] Qiao YL, Sellors JW, Eder PS, Bao YP, Lim JM (2008). A new HPV-DNA test for cervical-cancer screening in developing regions: a cross-sectional study of clinical accuracy in rural China. Lancet Oncol..

[7] Zhao PS, Liu SD, Zhong ZX, Hou JY, Lin LF (2018). Prevalence and genotype distribution of humanpapillomavirus infection among women in northeastern Guangdong Province of China. BMC Infect Dis.

[8] Xiao SS, Fan JL, He SL, Li YR, Wang LY (2016). Analysis of human papillomavirus infection in 16,320 patients from a gynecology clinic in central South China. J Low Genit Tract Dis.

[9] Li Z, Liu F, Cheng S, Shi L, Yan ZL (2016). Prevalence of HPV infection among 28,457 Chinese women in Yunnan Province, southwest China. Sci Rep.

[10] Sui S, Niyaz M, Zhu KC, Wang L, Lu P (2014). Significance of human papilloma virus subtype detection in oppor-tunistic screening for cervical cancer in Uygur and Han women. Chin J Clin Oncol.

[11] Herrero R, Hildesheim A, Bratti C, Sherman ME, Hutchinson M (2000). Population-based study ofhuman papillomavirus infection and cervical neoplasia in rural CostaRica. J Natl Cancer Inst.

[12] Rudolph SE, Lorincz A, Wheeler CM, Gravitt P, Ponce EL (2016). Population-based prevalence of cervical infection with human papillomavirus genotypes 16 and 18 and other high risk types in Tlaxcala. Mexico BMC Infect Dis.

[13] Cao D, Zhang SH, Zhang Q, Wei X, Zhao MY (2017). Prevalence of high-risk human papillomavirus infection among women in Shaanxi province of China: a hospital-based investigation. J Med Virol.

[14] Lee SJ, Cho YS, Cho MC, Shim JH, Lee KA (2001). Both E6 and E7 oncoproteins of humanpapillomavirus16inhibit IL-1 8induced IFN-gamma production in human peripheral bloodmononuclear and NK cells. J Immunol.

[15] Muñoz N, Franceschi S, Bosetti C, Moreno V, Herrero R (2002). International agency for research on cancer. multicentriccervical cancer study group. role of parity and humanpapillomavirus in cervical cancer: the IARC multicentric case-control study. Lancet.

[16] Alhamlan FS, Khayat HH, Ramisetty MS, Al-Muammar TA, Tulbah AM (2016). Sociodemographiccharacteristics and sexual behavior as risk factors for humanpapillomavirus infection in Saudi Arabia. Int J Infect Dis..

[17] Lekovich JP, Amrane S, Pangasa M, Pereira N, Frey MK (2015). Comparison of HumanPapillomavirus infection and cervical cytology in women usingcopper-containing levonorgestrel-containing intrauterine devices. Obstet Gynecol.

[18] Castellsagué X, Diaz M, Vaccarella S, Sanjosé SD, Muñoz N (2011). Intrauterine device use, cervical infection with human papillomavirus, and risk of cervical cancer: a pooled analysis of 26 epidemiological studies. Lancet Oncol.

[19] Rositch AF, Koshiol J, Hudgens MG, Razzaghi H, Backes DM (2013). Patterns of persistent genital human papillomavirus infection among women worldwide: a literature review and meta-analysis. Int J Cancer.

[20] Committee on Practice Bulletins—Gynecology (2012). ACOG practice bulletin number 131: Screening for cervical cancer. Obstet Gynecol.

[21] Zhang YR, Huang WH, Jing LP, Jing CX (2018). Influencing factors of persistent infection in some Area of Guangdong Province. J Sun Yat⁃Sen University(Medical Sciences).

[22] Zhang Q, Cao D, Ma Q, Li N, Cui XQ (2015). Natural outcome of genital tract high risk human papillomavirus infection and associated factors among 760 women. Zhongguo Yi Xue Ke Xue Yuan Xue Bao.

[23] Taylor S, Bunge E, Bakker M, Castellsagué X (2016). The incidence, clearance and persistence of non-cervical human papillomavirus infections: a systematic review of the literature. BMC Infect Dis.

[24] Guzalnur A, Peng ZL, Liu SL, Wang H (2004). The difference of expression of HPV and its subtypes among women from Northwest Sichuan and southern Xinjiang. Chin J Microbiol Immunol.

[25] Ramanakumar AV, Naud P, Roteli-Martins CM, Carvalho NS, Borba PC (2016). Incidence and duration of type-specific human papillomavirus infection in high-risk HPV-naïve women: results from the control arm of a phase II HPV-16/18 vaccine trial. BMJ Open.

[26] El-Zein M, Ramanakumar AV, Naud P, Roteli-Martins CM, Carvalho NS (2019). Determinants of acquisition and clearance of human papillomavirus infection in previously unexposed young womem. Sex Transm Dis.

[27] Weele PV, Logchem EV, Wolffs P, Broek IV, Feltkamp M (2016). Correlation between viral load, multiplicity of infection and persistence of infection in a Dutch cohort of young women. J clin Virol.

[28] Schmeink CE, Massuger LFAG, Lenselink CH, QuintW GV, Witte BI (2013). Prospective follow-up of 2,065 young unscreened women to study human papillomavirus incidence and clearance. Int J Cancer.

[29] Sammarco ML, Riccio ID, Tamburro M, Grasso GM, Ripabelli G (2013). Type-specific persistence and associated risk factors of human papillomavirus infections inwomen living in central Italy. Eur J Obs Gynecol.

[30] Smith EM, Johnson SR, Ritchie JM, Feddersen D, Wang D (2004). Persistent HPV infection in postmenopausal age women. Int J Gynaecol Obstet.

